# Government policy interventions to reduce veterinary antimicrobial consumption in production animals: a protocol for a systematic review and evidence map

**DOI:** 10.1186/s13643-025-02829-9

**Published:** 2025-06-03

**Authors:** Kayla Strong, Fiona Emdin, Sam Orubu, Susan Rogers Van Katwyk, Heather Ganshorn, Jeremy Grimshaw, Mathieu J. P. Poirier

**Affiliations:** 1 Global Strategy Lab, York University, North York, ON Canada; 2 Libraries and Cultural Resources, University of Calgary, Calgary, AB Canada; 3 Clinical Epidemiology Program, Ottawa Health Research Institute, Ottawa, ON Canada; 4 Department of Medicine, University of Ottawa, Ottawa, ON Canada; 5 School of Global Health, Faculty of Health, York University, North York, ON Canada

**Keywords:** AMR, Antimicrobial resistance, Policy, Government, Systematic review, Protocol

## Abstract

**Background:**

Globally, agricultural production systems consume two-thirds of all antimicrobials. These systems are used to raise animals that produce products for consumption, such as meat, eggs, milk, and wool. The World Bank estimates that by 2030, AMR will reduce global livestock production by up to 7.5%, resulting in economic losses of up to one trillion USD. Governments worldwide have implemented various policies to promote antimicrobial stewardship in production animals, such as requiring veterinary prescriptions for antimicrobial use, restricting certain antimicrobials, and prohibiting antimicrobial use for growth promotion. However, the efficacy of these measures remains uncertain, necessitating a comprehensive review to guide policymakers. This review will identify and describe implemented government policy interventions to reduce veterinary AMU and AMR in production animals. A secondary analysis will map the policy pathways and the stakeholders involved in their successful implementation.

**Methods:**

An electronic search strategy has been developed in consultation with a public health librarian and a veterinary health librarian. CAB Abstracts, MEDLINE, Web of Science, and ProQuest Dissertations will be searched, and additional studies will be identified using gray literature searches. The intervention of interest is any policy intervention enacted by a government or government agency in any country to change antimicrobial use in production animals. For inclusion within the review, studies must (1) describe the government policy, (2) quantitatively measure the impact of the policy in production animals using a rigorous study design, and (3) measure the impact of the intervention through antimicrobial use (AMU) or AMR. Two independent reviewers will screen for eligibility using defined criteria, and data will be extracted using Covidence software and Excel, respectively. Results will be synthesized narratively and visually (using maps and Sankey plots) to identify evidence gaps.

**Discussion:**

This systematic review is intended to inform future government policies addressing antimicrobial resistance and antimicrobial use in production animal systems. It will also inform future research priorities by identifying evidence gaps about the effectiveness of various policy interventions.

**Systematic review registration:**

Open Science framework.

**Supplementary Information:**

The online version contains supplementary material available at 10.1186/s13643-025-02829-9.

## Background

Antimicrobial resistance (AMR) occurs when bacteria, viruses, fungi, or parasites adapt to reduce or eliminate the effectiveness of antimicrobials [1]. Although AMR is a natural process, it has been accelerated by the underuse, misuse, and overuse of antimicrobials in human health and in agri-food systems leading to increasingly negative impacts on human health, food production, and other socioeconomic impacts such as reduced productivity or deepened economic inequalities between commercial and small-scale producers [2]. By 2030, AMR could reduce global animal production (which refers to systems used to raise animals that produce products for consumption such as meat, eggs, milk, and wool) by up to 7.5% and result in global economic losses of up to one trillion USD [1]. Antimicrobials are essential for animal welfare and production to treat and control infectious diseases [3]; however, prophylactic uses such as for growth-promotion [4] may contribute to the development of AMR [5].

Several antimicrobial classes used in veterinary medicine are the same as those used in human medicine, and resistance mechanisms can transfer within and between these sectors [6, 7]. Improving judicious and responsible veterinary AMU and reducing AMR in production animals will be essential to maintaining the effectiveness of antimicrobials for use in animals and humans and preserving the security of our food systems [8]. Recognizing the adverse outcomes accompanying the global spread of AMR, many countries have already implemented policies promoting the responsible and prudent use of antimicrobials in production animals [9, 10]. However, the impacts of these regulatory measures remain uncertain [11]. Evidence of effective policies that mitigate AMR risks associated with AMU in production animals is urgently needed.

To address the challenges of AMU and AMR in production animals, governments need evidence of what policy interventions are available, how they impact AMU and AMR, and how they could be applied. Currently, the World Organisation for Animal Health (WOAH, formerly OIE), the Food and Agriculture Organization (FAO), the World Health Organization (WHO) and the United Nations Environmental Program (UNEP) have a partnership called the Strategy on Antimicrobial Resistance and the Prudent Use of Antimicrobials to support governments, producers, and other stakeholders in moving towards the responsible use of antimicrobials in agriculture [12]. Although the partnership offers tools for addressing AMR in production animal systems, evaluations of these tools and governmental policies that stem from them are scarce. No reviews were found in the literature consolidating and weighing the benefits of different policy interventions targeting veterinary AMU and animal AMR in various production animals. There is a need for a systematic review that catalogs and assesses a broad set of government policy interventions that target veterinary AMU and production animal AMR across multiple contexts and settings.

The primary aim of this systematic evidence mapping project is to identify and describe government policy interventions intended to reduce veterinary AMU and AMR in production animals. A secondary analysis will map the policy pathways and the stakeholders involved in their successful implementation.

## Methods

This systematic review builds on the methods of a previously published systematic review and evidence map by Rogers Van Katwyk et al. (2019), which identified, described, and assessed government policy interventions to reduce human antimicrobial use [13]. A descriptive review and evidence mapping of existing pathways will be conducted.

**Fig. 1:**
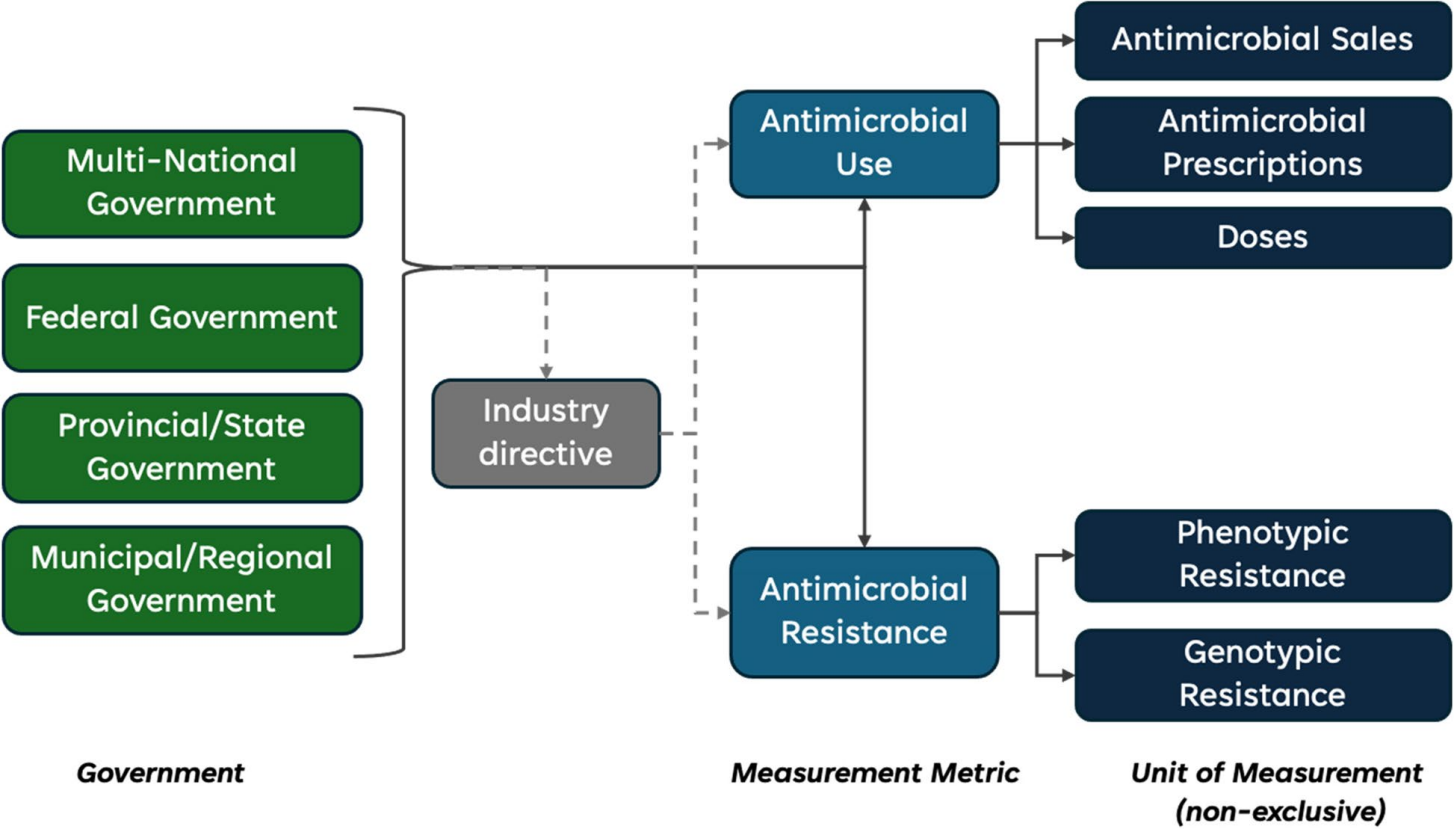
Placement. Measurements of interest for inclusion within the systematic review

This review will include studies if they report the impact of the policy on AMU or AMR in production animals (Fig. 1). Results specific to antimicrobial use and antimicrobial resistance will be interpreted separately. There are anticipated benefits to measuring each of these variables. AMU is anticipated to capture more studies and potentially a more representative sample than AMR studies, which are often constrained by data accessibility and the cost of resistance testing. Comparably, AMR studies can provide greater insight into these policies’ impact on resistance trends, which can be particularly helpful given the potential disconnect between prescribed antimicrobials versus those consumed.

We will define policy interventions by the Behavior Change Wheel framework as those that create change in antimicrobial use through education, persuasion, incentivization, coercion, training, restriction, changing the physical or social context, modeling appropriate behaviour, or reducing barriers to action [13]. Examples of government policy interventions that could be included in this study would be those regulating who can prescribe antibiotics to production animals (e.g., veterinarians only), restricting antibiotic use (e.g., banning antimicrobial use for growth promotion), or launching educational campaigns targeting specific producers (e.g., cattle feedlot producers).

### Protocol and registration

This study protocol was registered with the Open Science Framework (https://osf.io/56pzg) and is reported in line with the Preferred Reporting Items for Systematic Review and Meta-Analysis Protocols (PRISMA-P) guidelines. A populated PRISMA-P checklist is provided as an additional file (see Additional file 1). Changes from these protocols will be reported and discussed in the final manuscript.

### Criteria for including studies in this review

#### Study types to be included

To be included in the review, studies must quantitatively assess the impact of a government policy intervention measuring the change in (1) AMU or (2) AMR in production animals or animals that provide a product for eventual sale. Study designs may include, for example, interrupted time series, case–control, or before-after studies. All routes of antimicrobials addressed by the policy intervention are eligible for inclusion. These include but are not limited to oral, parenteral, or topical.

#### Types of participants

For this study, we defined production animals as any species of animals raised to produce products for consumption, including meat, eggs, milk, fur, leather, and wool. This will include commonly farmed species like avian, swine, bovine, caprine, camel, equine, rabbit, ovine, fish, bees, molluscs, mink, ferrets, and crustacean species, as well as other aquatically farmed species but can include less commonly farmed groups like other rodents and other aquatic species of animals. There are no spatial or temporal limitations to be included.

#### Types of interventions

We will include studies assessing the impact of any policy intervention type enacted by a government to affect the production of animal antimicrobial use or antimicrobial resistance in the production of animal species. Any level of government intervention, including those enacted by national, state, provincial, regional, and municipal governments, or by a government-controlled agency, ministry, or department, is eligible for inclusion. We will also include interventions where governing bodies may have partnered with an international organization to implement an initiative. We will include population-based policy interventions (e.g., targeting all swine in a nation) or policies targeting specific stakeholders, such as swine veterinarians or farmers. Interventions developed by an industry, pharmaceutical company, or veterinary organization independent from governing bodies will not be eligible for inclusion.

#### Types of comparisons

To ensure we capture the equally broad range of government policy interventions that have been implemented and evaluated, our review will include all quantitative study designs. Table 3 in the Appendix summarizes the inclusion and exclusion criteria.

#### Types of outcomes

We will include studies that evaluate a government policy at any level: from ecological (e.g., populations across multiple regions or one country) to the single farm or single group (herd, flock, barn, or pen) level. Studies may use data collected from anyone involved in selling, purchasing, or prescribing an antimicrobial drug, such as veterinarians, livestock producers, insurers, or regulatory agencies. Studies measuring antimicrobial consumption may use any measurement of antimicrobial use (e.g., mg/kg, mg of active ingredient, daily defined doses, or mg/population correction unit (PCU)) in any production animal population. Antimicrobial use may be described in terms of sales (the amount of antimicrobials purchased), antimicrobials prescribed (how many antimicrobials are ordered for treatment), or how much antimicrobials are administered (how much antimicrobial is being given to the animal). Additional antimicrobial use measurements will also be accepted.

AMR studies can measure either phenotypic resistance (e.g., the prevalence of bacterial isolates with a breakpoint indicating resistance) or genotypic resistance (e.g., the prevalence of bacteria carrying a resistance gene of interest). Data collected on absolute or relative change will be included to interpret the impact of government policy interventions.

#### Information sources

As part of this systematic review, four electronic databases will be searched: CAB Abstracts, MEDLINE, Web of Science, and ProQuest Dissertations PubMed (MEDLINE) from inception to the time of the search. The MEDLINE search strategy is available in Table 1. The original search strategy was derived from a previously published systematic review and meta-analysis examining the restricted use of antibiotics in food-producing animals and its associations with antibiotic resistance in food-producing animals and human beings [14]. Since the question this strategy is attempting to answer is more narrow in scope than our own, we adjusted this strategy to capture more policy types. Previously identified articles that were deemed relevant for inclusion in the systematic review were identified as indicator articles and their inclusion verified in trial runs of the search strategy [15–17]. Antibiotics included in this search strategy are derived from WOAH’s list of antimicrobials important to veterinary medicine [18]. This search strategy was designed by an information specialist and a librarian after consultation with veterinary and production animal experts. We are not applying date requirements. Articles will be accepted if they are written in one of the six official languages of the UN (Arabic, Chinese, English, French, Russian, and Spanish).

**Table 1:** MEDLINE search strategy for this systematic review

Theme	Identifier	Searches
**Antibiotic use and antimicrobial resistance**	1	Anti-bacterial agents/
	2	Anti-infective agents/
	3	1 or 2
	4	((antibacterial or anti-bacterial or antibiotic or anti-biotic or antimicrobial or anti-microbial) adj2 (resistan* or susceptib* or minimum inhibitory concentration)).kw,tw
	5	((aldesulfone or amdinopenicillin* or amikacin or aminocyclitol* or aminoglycoside* or aminopenicillin* or amoxicillin* or ampicillin or amphenicol* or ansamycin* or antipseudomonal or antistaphylococcal or apramycin or arbekacin or aspoxicillin or avilamycin or avoparcin or azalide or azidocillin or azithromycin or azlocillin or aztreonam or bacampicillin or bacitracin or baquiloprim or bekanamycin or benzylpenicillin or biapenem or bicozamycin or bicyclomycin* or brodimoprim or calcium aminosalicylate or capreomycin or carbadox or carbapenem* or carbenicillin or carboxypenicillin* or carindacillin or carumonam or cef* or cepha* or chloramphenicol or chlortetracycline or cinoxacin or ciprofloxacin or clarithromycin or clindamycin or clofazimine or clometocillin or clomocycline or cloxacillin or colistin or cyclic ester* or cyclic polypeptide* or cycloserine or dalbavancin or dalfopristin or danofloxacin or dapsone or daptomycin or demeclocycline or diaminopyrimidine* or dibekacin or dicloxacillin or difloxacin or dirithromycin or dihydrostreptomycin or doripenem or doxycycline or dihydrofolate reductase inhibitor* or enoxacin or enramycin or enrofloxacin or epicillin or ertapenem or erythromycin or ethambutol or ethionamide or faropenem or fleroxacin or flomoxef or florphenicol or flucloxacillin or flumeqin* or fluoroquinolone* or flurithromycin or fosfomycin or framycetin or furaltadone or furazolidone or fusidic acid or gamithromycin or garenoxacin or gatifloxacin or gemifloxacin or gentamicin or glycopeptide* or glycylcycline* or gramicidin or grepafloxacin or hetacillin or ibafloxacin or iclaprim or imipenem or ionophore* or isepamicin or isoniazid or josamycin or kanamycin or ketolilde* or kitasamycin or lasalocid or latamoxef or levofloxacin or lincomycin or lincosamide* or linezolid or lipopeptide* or lomefloxacin or loracarbef or lymecycline or macrolide* or maduramycin or marbofloxacin or mecillinam or meropenem or metacycline or metampicillin or methicillin or meticillin or metronidazole or mezlocillin or midecamycin or miloxacin or miocamycin or minocycline or mirosamycin or monensin or monobactam* or morinamide or moxifloxacin or mupirocin or nafcillin or nalidixic acid or narasin or neomycin or netilmicin or nifurtoinol or nitrofur* or nitroimidazole* or norfloxacin or novobiocin or ofloxacin or oleandomycin or orbifloxacin or oritavancin or ormosulfathiazole or ornidazole or orthosomycin* or oxacillin or oxazolidinone* or oxolinic acid or oxytetracycline or panipenem or para-aminosalicylic acid or paromomycin or pazufloxacin or pefloxacin or penamecillin or penethatamate or penicillin* or penimepicycline or phenethicillin or pheneticillin or phenicol* or phenoxypenicillin* or phenoxymethylpenicillin or phthalylsulfathiazole or pipemidic acid or piperacillin or pirlimycin or piromidic acid or pivampicillin or pivmecillinam or pleuromutilin* or polymixin* or polymyxin* or polypeptide* or pristinamycin or propicillin or protionamide or prulifloxacin or pseudomonic acid* or pyrazinamide or pyrimethamine or quinolone* or quinoxaline* or quinupristin or retapamulin or ribostamycin or rifa* or riminofenazine* or rokitamycin or rolitetracycline or rosoxacin or roxithromycin or rufloxacin or salfadoxine or salinomycin or semduramicin or sisomicin or sitafloxacin or sodium aminosalicylate or sparfloxacin or spectinomycin or spiramycin or streptoduocin or streptogramin* or streptomycin or sulbenicillin or sulfachlorpyridazine or sulfadi* or sulphadi* or sulfafurazole or sulphafurazole or sulfaisodimidine or sulfisoxazole or sulfon*or sulphon* or sulfaguanidine or sulfam* or sulfanilamide or sulfalene or sulfam* or sulpham* or sulfanilamide or sulfap* or sulphap* or sulfaquinoxaline or sulfath* sulphath* or sultamicillin or talampicillin or teicoplanin or telavancin or telithromycin or temafloxacin or temocillin or terdecamysin or terizidone or tetracycline* or tetroxoprim or thiamphenicol or tiamulin or ticarcillin or tigecycline or tildipirosin or tilmicosin or tinidazole or tiocarlide or tobicillin or tobramycin or trimethoprim or troleandomycin or trovafloxacin or tulathromycin or tylosin or tylvalosin or valnemulin or vancomycin or virginiamycin) adj2 (resistan* or susceptib* or minimum inhibitory concentration)).kw,tw
	6	4 or 5
	7	3 or 6
	8	exp Drug Resistance, Microbial/
	9	exp Anti-Bacterial Agents/or exp Animal Feed/
	10	((antibiotic? or anti-biotic? or abx or antibacterial? or anti-bacterial? or antifungal? or anti-fungal? or antimicrobial? or anti-microbial? or antiviral? or anti-viral? or bacterial? or microbial?) adj5 (resistan* or nonsusceptib* or non-susceptib*)).tw,kw,kf
	11	(antibacterial or anti-bacterial or antibiotic or anti-biotic or anti-microbial or antimicrobial).kw,tw
	12	(AMR adj10 resistan*).tw,kw,kf
	13	8 or 9 or 10 or 11 or 12
**Change/Impact**	14	(adjust* or alter* or change or changes or changing or control* or decreas* or limit* or modify or modified or modifying or reduce or reducing or reduction* or restrict* or appropriate or inappropriate or rational* or irrational*).ab,ti
	15	((reduc* or decreas* or restrict* or limit* or ban or bans or banning or eliminat* or control* or regulat* or less* or cut* or scale* or scaling or down* or taper*) adj5 ("use"or usage or utilization or dose* or dosage or administ* or prescri*)).kw,tw
	16	14 or 15
**Policy**	17	exp policy/
	18	exp policy making/
	19	exp legislation as Topic/
	20	exp Government/
	21	Government Regulation/
	22	(government* or international or multi?national or multi?lateral or national or federal or state or provinc* or municip* or region* or district* or department* or ministr* or agenc* or organi#ation*).kf,tw
	23	20 or 21 or 22
	24	(program* or campaign* or policy or policies or guideline* or strateg* or ban or banned or Regulat* or law or laws or prohibit* or restrict* or legislat* or report* or tax* or audit* or formular* or expenditure* or spending or label* or market* or advertis* or consultation* or import* or export*).ab,ti
	25	(health$ adj2 (policy or policies or planning or priorit$)).ab,ti
	26	18 or 19 or 24 or 25
	27	23 and 26
	28	7 and 13 and 16 and 27
**Production Animals**	29	exp animals/not humans.sh
	30	exp Poultry/
	31	exp Ruminants/
	32	exp Swine/
	33	exp Bees/
	34	exp Seafood/
	35	exp Mollusca/
	36	exp Crustacea/
	37	(food animal* or farm* or production animal* or livestock or feedlot* or animal feeding operation* or AFO or CAFO).kw,tw
	38	(ruminant* or cattle or bovine or cow* or beef or heifer* or steer* or calf or calves or sheep or ovine or caprine or goat* or equine or horse* or lepine or rabbit* or deer or elk or game or buffalo or bison or swine or pork or pig* or hog* or boar*).kw,tw
	39	(chicken* or broiler* or turkey* or duck* or geese or goose or poultry or fowl or avian).kw,tw
	40	(bee or bees or honeybee* or apiary or apicultur*).kw,tw
	41	((farm* or aquaculture) adj2 (fish or shellfish or seafood or amberjack or arapaima or asp or atipa or barb or barramundi or bass or beluga or bluefin or bluefish or bocachico or bonythongue or bream or bullhead or carp or catfish or char or cichlid or cobia or cod or dorada or eel* or gourami or guapote or grouper or halibut or lai or loach or mackerel or mandarin fish or meagre fish or milkfish or mojarra or mullet or mudfish or nori nei or perch or pejerrey or pike or porgy or pompano or red drum or roach or roho labeo or salmon or sampa or seabass or seabream or snakehead or snapper or snook or sole or spinefood or sturgeon or sweetfish or tench or tilapia or trout or tuna or turbot or vendace or whitefish)).kw,tw
	42	((farm* or aquaculture) adj2 (shrimp or prawn* or crayfish or lobster* or crab*)).kw,tw
	43	((farm* or aquaculture) adj2 (abalone or bivalve* or clam* or carpet shell or cockle* or corbicula or geoduck or mussel* or oyster* or periwinkle* or quahog or sand gaper* or scallop* or shellfish or tagelus or venus)).kw,tw
	44	aquaculture.kw,tw
	45	Aquaculture/
	46	26 or 28 or 29 or 30 or 31 or 32 or 33 or 34 or 35 or 36 or 37 or 38 or 39 or 40 or 41 or 42 or 43 or 44 or 45
	47	28 and 46

Gray literature will also be included and will be identified through targeted web searching, specifically searching the World Bank, UN, WHO, and their subsidiary organization websites, by searching dissertation databases and by searching references of included studies to identify non-indexed articles. We will contact subject-matter experts from the World Organization of Animal Health and other international production animal organizations to identify additional studies that fit our inclusion criteria.

#### Screening and eligibility

Article screening will occur in two stages: (1) title and abstract and (2) full text. The two stages will ensure studies are captured that might not indicate they are measuring outcomes of a government policy intervention within their title and abstract. Within the title and abstract screening, studies will be assessed if they are empirical, specific to production animals, and if they measure AMU or AMR. Later, at full-text review, the articles will be more thoroughly assessed to determine if they meet additional inclusion/exclusion criteria, including whether the policy intervention examined is a government policy intervention (Table 3 in Appendix). A previously published systematic review of human government interventions targeting AMU screened 13,635 abstracts and identified 69 studies. Similarly, we are expecting to include many studies in the title and abstract (15,000–20,000) and end up including 50–70 studies.

We have tested our inclusion criteria on a random subset of records (200 studies) in both title and abstract and full-text screening and had two reviewers apply the criteria independently to check for consistency. We did not find the need to adjust the criteria from what is described in these methods. We will resolve any future discrepancies through consensus-based discussions.

#### Data management and extraction

Two reviewers will independently complete the title and abstract screening, full-text screening, data extraction, and risk of bias assessment. Reviewer disagreements will be resolved by a third reviewer. A PRISMA chart will be used to display the flow of studies included during different stages of the review. Screening and data extraction will be completed using Covidence and Microsoft Excel.

#### Types of outcomes

The primary outcome is the type of government intervention and whether the intervention was successful when evaluating either of the two measurements of interest (AMU and AMR in production animals). All types of interventions, and key conclusions from the authors regarding the policies’ effectiveness will be recorded. If the effectiveness of the intervention changes over time or over different areas, the spatial and temporal variations in the interventions’ impact will be recorded.

Any other beneficial or negative impacts related to the governmental intervention will be recorded as secondary outcomes.

A summary of key data extraction fields can be found in Table 2. Two authors tested these data extraction fields on two studies known to be eligible for inclusion and adjusted the extraction criteria as needed. Specifically, we added extraction fields for classifying the policy intervention (using the Behavior Change Wheel (e.g., legislation or regulation) by primary target of the policy intervention (e.g., antimicrobial guidelines, interventions banning certain types, or uses of antimicrobials). Since this systematic review captures papers with multiple outcomes, we have also added additional data collection fields for the type of outcome being examined by the study and how this outcome was assessed by study authors (e.g., sampling methods if looking at resistance samples).

**Table 2:** Data extraction fields for the systematic review of government policy interventions to reduce veterinary antimicrobial consumption in production animals

**General information**	**Last name, first author, year of publication**
	Primary contact author’s email
	Study design (i.e., pre-post, DID, ITS)
	Primary country
	Study aim
	Ethics approval
	Funding source
	Meets PICOS requirements (Y/N)
	Reason to exclude
**Intervention Details**	Government intervention(s)—be as detailed as possible (delivery method, etc.)
	Non-governmental co-interventions
	Intervention’s target (i.e., specific antimicrobials like growth promoters, animal type, veterinary prescriptions, medically important antimicrobials)
	Intervention date(s)
	Source of data (or describe primary collection)
	Dates captured by study
	Enacting government
	Primary policy type – Behavior Change Wheel
	Primary policy type – specific (e.g., banning certain types of antimicrobials, banning certain uses of antimicrobials)
	Secondary policy targets
	Policy government level (e.g., national, provincial/state, regional)
**Intervention impact**	Outcome measure (i.e., animal AMU, animal AMR, human AMR)
	How the outcome measure is assessed (sampling method, etc.)
	Key conclusions of the study authors – impact of the intervention
	Meaningful difference if reported AND measure of significance (if provided)
**Secondary outcomes**	Secondary outcomes: beneficial
	Secondary outcomes: adverse
	Miscellaneous comments from the study authors or by the review authors

#### Missing data

Missing data will be noted on extraction forms. Authors will be contacted twice for any missing data they might inform the descriptive review using corresponding author emails or emails found online.

#### Risk of bias

Risk of bias will be assessed at the study level using two RoB tools: the Cochrane Risk of Bias tool for randomised trials [19] and RoBANS 2 [20] for observational studies. Both tools classify studies as having an overall low, high, or unclear risk of bias across several domains. Consensus will resolve disagreements between reviewers. Results will be displayed in a summary table.

#### Interpretation of review findings

To make recommendations, the quality of evidence across included studies will be assessed using the Grading of Recommendations Assessment, Development and Evaluation (GRADE) system, and findings will be presented in a summary table. We will follow previous recommendations on how to apply GRADE when a meta-analysis is not performed [21].

### Strategy for analysis

#### Descriptive review

Data will be summarized narratively and in a table that displays details on all studies. Studies will be categorized by policy approach (or policy category) as defined by the Behavior Change Wheel framework as a communication/marketing, guideline, fiscal, regulation, legislative, environmental/social planning, or service provision initiative [22]. Studies will also be classified by policy option (e.g., policies banning antimicrobial types in certain production species, policies banning types of antimicrobial use in production animals) and by the government level which enacted them (e.g., federal, state, provincial, or municipal) and according to study design (e.g., randomized controlled trial, non-randomized controlled before and after design, uncontrolled before and after design, time series design, cohort design, descriptive design). Studies will be mapped visually by the relationships between these factors. A secondary evidence map will consider policies’ governance pathways to reach production animals. There is particular interest in the role industry has in connecting governmental policies to action within production animal systems, and a summary map will be developed to show these conduits. A descriptive review will be supported by a discussion on the range of effects, heterogeneity, and the quality of evidence.

## Discussion

Although many countries are already promoting responsible and prudent AMUs by international standards, little guidance or research is available to support policymakers. No global government policy reviews target AMU and AMR across all production animal systems. This project will fill this knowledge gap and can guide policymakers who wish to develop, enact, and plan policies targeting AMU and AMR in animal production.

### Strengths and limitations

This review has several notable strengths. Firstly, its methods build on a previously published, rigorous, and reproducible methodology [13]. Secondly, it is being conducted by a cross-disciplinary team that brings expertise across the One Health continuum, prioritizing bringing a One Health perspective to the review. Thirdly, it encompasses a broad search string and inclusion criteria when selecting production animals, allowing a more holistic interpretation of existing policies.

The study also has several potential limitations, including publication bias, confounding variables, and heterogeneity. We discuss these limitations and methods to address them.

Publication bias may sway our analysis away from studies that report government policy initiatives with null results and overestimate the proportion of interventions causing significant changes in AMR or AMU. Search strategies that capture academic publications may bias selection towards only specific study types; for example, our search strategy might select for studies from high-income countries that may not be generalizable to the global context. Given the scope of the review and the limited language expertise of our team, we are unable to conduct systematic searches in languages other than English. To mitigate these limitations, the search strategy for this paper was designed specifically to search across multiple sources, including gray literature sources, and uses expert consultation to identify additional relevant research, which may be more likely to capture initiatives with null results and include perspectives from global contexts. Although our search strategy was developed in English, we will include studies written in any of the UN’s six official languages (Arabic, Chinese, English, French, Russian, and Spanish) if they are identified through our search process.

Heterogeneity refers to variability between studies. Given the breadth of studies intended for inclusion in this review, there is likely to be substantial heterogeneity between different studies, making studies unsuitable for meta-analysis. For this reason, we are not planning to pursue a meta-analysis at this time; if sufficient studies are appropriate for comparison, given similarities in intervention, outcome measurements, and study design, meta-analysis can be pursued in future studies.

In studies where antimicrobial resistance is an outcome, there is an additional limitation: some changes in antimicrobial resistance may be delayed relative to when a policy change is introduced, such as if there is an established resistance reservoir or limited fitness cost. This will be discussed as a potential limitation when interpreting findings.

## Supplementary Information

Additional file 1. PRISMA-P Checklist. Populated PRISMA-P checklist for study protocol.

## Data Availability

Not applicable.

## Appendix

**Table 3:** Inclusion and exclusion criteria for title and abstract screening and for full text screening phases

	**Criteria**	**Include If**	**Exclude If**
**Title and abstract screening**	The study assesses a population of interest	The study reports on a population of production animals (defined as animals raised to produce products for consumption including avian, swine, bovine, caprine, camel, equine, rabbit, ovine, fish, bees, molluscs, mink, ferrets, and crustacean species) at an individual, group (herd, flock, pen, barn) or population level	The study only considers companion animals
	The study assesses a primary outcome measure of interest	The study reports on either: 1) change in antimicrobial use in production animals including antimicrobial administered (how much antimicrobial is being given to the animal), antimicrobial prescribing (how much antimicrobials are ordered for treatment), and/or antimicrobial sales (the amount of antimicrobials purchased). Additional antimicrobial use measurements will also be accepted 2) change in antimicrobial resistance in production animals including absolute or relative change in phenotypic or genotypic resistance *All routes of antimicrobial administration are eligible for inclusion*	The study does not report on antimicrobial use or antimicrobial resistance in production animals
	The study is empirical research	The study is empirical research	The study is qualitative or theoretical, including editorials, reviews, and commentaries
	A policy intervention is evaluated	A policy intervention is evaluated	Intervention does not evaluate a change to a policy
**Full text screening**	The study assesses a population of interest	The study reports on a population of production animals (defined as animals raised to produce products for consumption including avian, swine, bovine, caprine, camel, equine, rabbit, ovine, fish, bees, molluscs, mink, ferrets, and crustacean species) at an individual, group (herd, flock, pen, or barn) or population level	The study only considers companion animals
	The study assesses a primary outcome measure of interest	The study reports on either: 1) change in antimicrobial use in production animals including antimicrobial administered (how much antimicrobial is being given to the animal), antimicrobial prescribing (how much antimicrobials are ordered for treatment), and/or antimicrobial sales (the amount of antimicrobials purchased). Additional antimicrobial use measurements will also be accepted 2) change in antimicrobial resistance in production animals including absolute or relative change in phenotypic or genotypic resistance *All routes of antimicrobial administration are eligible for inclusion*	The study does not report on antimicrobial use or antimicrobial resistance in production animals
	The study quantitatively evaluates the effect of an intervention	The study quantitatively evaluates the effect of an intervention	The study is theoretical (i.e. an editorial, a commentary), a narrative review, or does not evaluate the impact of an intervention
	A government policy intervention is evaluated	The policy intervention was enacted by a government or government agency at the federal, state, provincial, or municipal level	Policy intervention was not enacted by a government or without government recommendation. I.e., a policy was enacted by an industry with no government involvement
	The intervention is clearly described	The study provides a description that includes the intervention’s aim, enacting government authority, timing, and policy type	The study does not describe the aim, governing body, timing of the intervention, or policy type of the intervention
